# Systematic review of disease-modifying antirheumatic drugs for juvenile idiopathic arthritis

**DOI:** 10.1186/1471-2431-12-29

**Published:** 2012-03-15

**Authors:** Alex R Kemper, Heather A Van Mater, Remy R Coeytaux, John W Williams, Gillian D Sanders

**Affiliations:** 1 Department of Pediatrics, Duke University School of Medicine, Durham, NC, USA; 2 Duke Evidence-based Practice Center, Duke Clinical Research Institute, Duke University School of Medicine, Durham, NC, USA; 3 Department of Community and Family Medicine, Duke University School of Medicine, Durham, NC, USA; 4 Department of Medicine, Duke University School of Medicine, Durham, NC, USA; 5 Center for Health Services Research In Primary Care, Durham VA Medical Center, Durham, NC, USA

**Keywords:** Juvenile rheumatoid arthritis, Disease-modifying antirheumatic drugs, Comparative effectiveness research, Systematic review

## Abstract

**Background:**

Treatment of juvenile idiopathic arthritis (JIA) with disease-modifying antirheumatic drugs (DMARDs) may improve outcomes compared to conventional therapy (e.g., non-steroidal anti-inflammatory drugs, intra-articular corticosteroids). The purpose of this systematic review was to evaluate the comparative effectiveness and safety of DMARDs versus conventional therapy and versus other DMARDs.

**Results:**

A systematic evidence review of 156 reports identified in MEDLINE^®^, EMBASE^®^, and by hand searches. There is some evidence that methotrexate is superior to conventional therapy. Among children who have responded to a biologic DMARD, randomized discontinuation trials suggest that continued treatment decreases the risk of having a flare. However, these studies evaluated DMARDs with different mechanisms of action (abatacept, adalimumab, anakinra, etanercept, intravenous immunoglobulin, tocilizumab) and used varying comparators and follow-up periods. Rates of serious adverse events are similar between DMARDs and placebo in published trials. This review identified 11 incident cases of cancer among several thousand children treated with one or more DMARD.

**Conclusions:**

Few data are available to evaluate the comparative effectiveness of either specific DMARDs or general classes of DMARDs. However, based on the overall number, quality, and consistency of studies, there is moderate strength of evidence to support that DMARDs improve JIA-associated symptoms. Limited data suggest that short-term risk of cancer is low.

## Background

Non-steroidal anti-inflammatory drugs (NSAIDs) and corticosteroids (systemic or intra-articular) are only partially effective in treating the symptoms of juvenile idiopathic arthritis (JIA) and its long-term complications. Treatment with disease-modifying antirheumatic drugs (DMARDs) are increasingly used because they appear to lead to better disease control. DMARDs, which interfere directly with immune cells or their function to reduce inflammation, are typically classified as either biologic (i.e., created by biologic processes) or non-biologic drugs, also referred to as synthetic DMARDs. Table 1 lists the commonly used DMARDs, their mechanism of action, and whether they have been approved for use in children by the U.S. Food and Drug Administration (FDA). Methotrexate is often considered a component of conventional treatment, along with NSAIDs and corticosteroids.

**Table 1:** DMARDs Evaluated

Generic name	Mechanism of action	FDA-approved for JIA?*
Biologic		

Abatacept	T-cell co-stimulation modulator; soluble fusion protein	Yes

Adalimumab	TNF inhibitor; anti-TNF monoclonal antibody	Yes

Anakinra	IL-1 receptor antagonist	No

Canakinumab	IL-1 inhibitor; anti-IL-1beta monoclonal antibody	No

Etanercept	TNF inhibitor; fusion protein TNF receptor inhibitor,	Yes

Infliximab	TNF inhibitor; anti-TNF monoclonal chimeric antibody	No

IVIG	Interaction with activating Fc receptors	No

Rilonacept	IL-1 inhibitory; soluble fusion protein	No

Rituximab	Binds to CD20 antigen	No

Tocilizumab	IL-6 receptor antagonist	No

Non-Biologic		

Azathioprine	Purine synthesis inhibitor	No

Cyclosporine A	Calcineurin inhibitor	No

Penicillamine	Unknown (may lower IgM rheumatoid factor, depresses T-cell activity)	No

Hydroxy-chloroquine	Not well understood, may reduce T-lymphocyte transformation and chemotaxis	No

Leflunomide	Isoxazole immunomodulatory agent	No

Methotrexate	Unknown (anti-metabolite, inhibits dihydrofolic acid reductase)	Yes

Mycophenolate mofetil	Guanosine synthesis inhibitor	No

Sulfasalazine	Unknown	Yes

Tacrolimus (FK506)	Calcineurin inhibitor	No

Thalidomide	Unknown	No

*Labeling refers to any pediatric approval

*Abbreviations: CD *cluster of differentiation, *Fc *fragment crystallizable, *FDA *U.S. Food and Drug Administration, *IgM *immunoglobulin M, *IL *interleukin, *IVIG *intravenous immunoglobulin, *JIA *juvenile idiopathic arthritis, *T-cell/-lymphocyte *thymus cell/lymphocyte, *TNF *tumor necrosis factor

To inform clinicians, patients, and families about the evidence regarding the management of JIA with DMARDs, and to help researchers identify critical gaps in knowledge, the Agency for Healthcare Research and Quality (AHRQ) commissioned a comparative effectiveness review (CER) [1]. This report summarizes the central findings from that CER review about the effects of DMARDs in children with JIA. Because treatment and outcomes can vary by category of JIA, we attempted to separately analyze these when data were available, following the International League of Associations for Rheumatology classification (i.e., systemic arthritis, oligoarthritis, rheumatoid factor-negative polyarthritis, rheumatoid factor-positive polyarthritis, psoriatic arthritis, enthesitis-related arthritis, undifferentiated JIA) [2].

Safety is an especially important concern because the FDA has placed a box warning on the DMARDs that target tumor necrosis factor alpha (TNF-α) (e.g., etanercept, infliximab, adalimumab), because of concerns about increased risk of malignancy. Although there are other potential safety issues, we focus in this summary report only on the risk of cancer and death.

In this summary, we address the following key questions:

1. Does treatment with DMARDs compared to conventional treatment with or without methotrexate improve laboratory measures of inflammation, radiological progression, symptoms, or health status?

2. What are the comparative effects of different DMARDs on these health outcomes?

3. Do the rate and type of serious adverse events differ between DMARDs or between DMARDs and conventional treatment with or without methotrexate?

4. How do the efficacy, effectiveness, and safety of treatment with DMARDs differ across categories of JIA?

## Methods

### Search strategy and identification of relevant studies

We searched MEDLINE^® ^(1966 through December 2010) and EMBASE^® ^(1947 through December 2010) using Medical Subject Headings (MeSH) terms and key words for JIA and its older designations (i.e., juvenile rheumatoid arthritis [JRA], juvenile chronic arthritis [JCA]), the generic and brand names DMARDs, and the names of instruments used to assess outcomes. The complete search strategy is available in the CER [1]. The search was limited to English-language reports of human studies. These searches were supplemented by review of the bibliographies of included studies and searches for publications of potentially eligible studies from abstracts presented in 2008 and 2009 at meetings of the American College of Rheumatology (ACR), the European League Against Rheumatism, and the Pediatric Academic Societies.

### Study selection

For efficacy, we included studies with a sample population of individuals 18 years or younger with JIA treatment for at least 3 months that included a comparator. To better understand the potential risk for serious adverse events, we also included case reports and letters to the editor. Two independent reviewers reviewed all abstracts for potential inclusion. The full texts of all potentially eligible reports were evaluated for inclusion by two independent reviewers. Differences were resolved by consensus.

### Data extraction

Abstractors worked in pairs: the first abstracted the data, and the second over-read the article and the abstraction to assure accuracy. To evaluate response to therapy, disease activity, and functional status, we abstracted data from commonly used measures including the ACR Pediatric 30, 50, 70, 90, or 100 response [3], active joint count, time to flare, remission or inactive disease, the physician global assessment of disease activity by visual analog scale (0 to 100 mm, with higher scores indicating greater disease activity), parent or patient global assessment of well-being by visual analog scale, and the Childhood Health Assessment Questionnaire (CHAQ), which measures functional ability [4]. For trials that evaluated efficacy, the abstractors also assessed study quality, generating a summary rating of good, fair, or poor. These ratings considered allocation, blinding, outcome assessment, and follow-up, using methods defined by AHRQ [5]. The rating and corresponding rationale for each study is available in the CER [1].

## Results

### Literature search and screening

**Figure 1:**
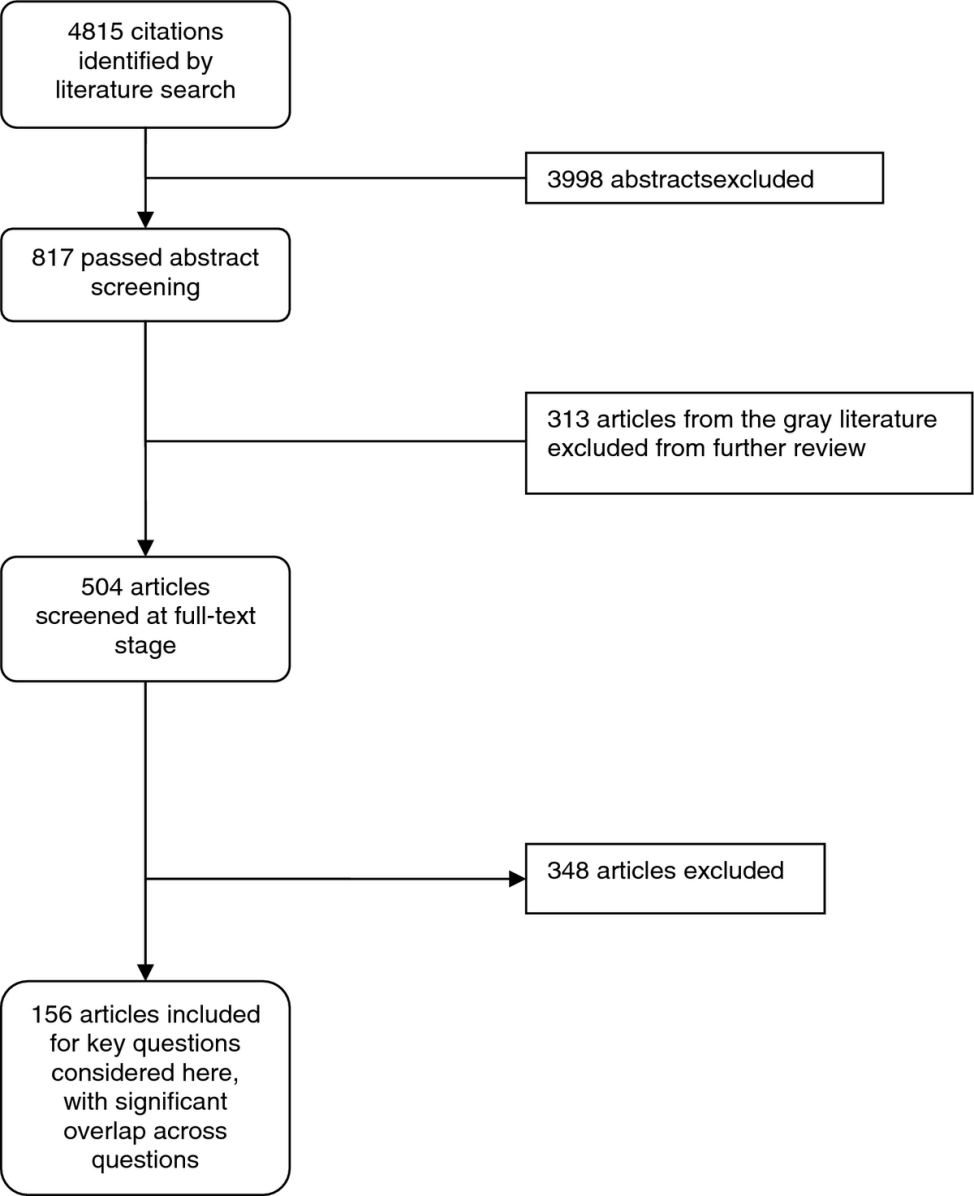
**Literature flow diagram**. This figure describes the flow of literature for the original AHRQ-sponsored CER, which included one key question not considered in the present report. Citations were not separated out by key question until the full-text screening stage. Reasons for exclusion are available in Appendix F of Reference 1.

**Figure 2:**
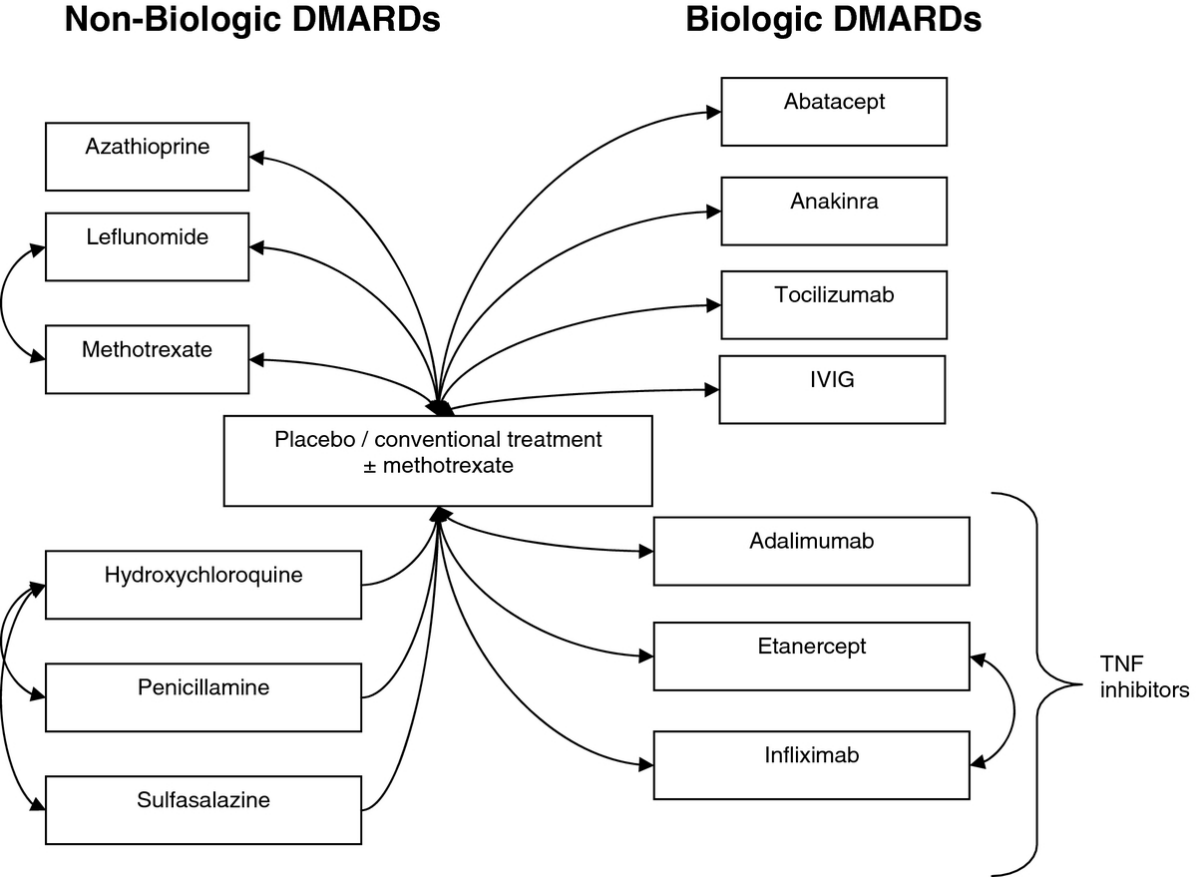
**Treatment comparisons evaluated in the efficacy studies**.

We identified 4815 citations, of which 156 met eligibility criteria for at least one of the key questions. Figure 1 illustrates the selection process. Figure 2 summarizes the treatment comparisons from the included efficacy studies. Six non-biologic DMARDs and seven biologic DMARDs have been compared to conventional treatment with or without methotrexate. Three different sets of non-biologic DMARDs have been directly compared (leflunomide vs. methotrexate, hydroxychloroquine vs. penicillamine, and hydroxychloroquine vs. sulfasalazine), and two biologic DMARDs have been directly compared (etanercept vs. infliximab). Because gold is rarely used in the treatment of JIA, we did not consider it among the therapies. However, we do describe its use in the included studies. Three of the biologic DMARDs that have been compared to conventional treatment were in the same class (TNF-α inhibitors: adalimumab, etanercept, and infliximab). Study heterogeneity precluded meta-analysis of this combined class versus conventional treatment.

## DMARDs vs. Conventional therapy (key question 1)

### Biologic DMARDs vs. Conventional treatment with or without Methotrexate

#### Abatacept

One good-quality randomized discontinuation study evaluated abatacept in children with persistent oligoarthritis, extended oligoarthritis, polyarthritis, or systemic JIA [6]. During the 6-month double-blind period of this study, there was statistically significant improvement compared to placebo in the active joint count (4.4 vs. 6; p = 0.02), CHAQ score (0.8 vs. 0.7; p = 0.04), physician global assessment (14.7 vs. 12.5; p < 0.01), and ACR Pediatric 90 response (40% vs. 16%; p < 0.01). There was no statistically significant improvement in parent or patient global assessment (17.9 vs. 23.9; p = 0.70) or erythrocyte sedimentation rate (ESR; 25.1 vs. 30.7; p = 0.96).

#### Adalimumab

One good-quality randomized discontinuation trial compared adalimumab to conventional therapy among children with polyarticular JRA [7]. The results were stratified by use of methotrexate. At the end of the 48-week double-blind phase, fewer patients treated with adalimumab and methotrexate had flares than those treated with placebo plus methotrexate (43% vs. 71%; p = 0.03). Similarly, the proportion of patients who had a flare of disease in the adalimumab without methotrexate group was lower than in the placebo group without methotrexate (37% vs. 65%; p = 0.02). The ACR Pediatric 50 response in the adalimumab without methotrexate group was higher than in the placebo without methotrexate group (53% vs. 32%; p = 0.01), and higher than in those groups that received methotrexate (63% vs. 38%; p = 0.03). Although the ACR Pediatric 90 response was higher in the adalimumab without methotrexate group than in the placebo without methotrexate group (30% vs. 18%), the difference was not statistically significant (p = 0.28). Similarly, the ACR Pediatric 90 response among those who also received methotrexate was higher in the adalimumab group than in the placebo group, but did not achieve statistical significance (42% vs. 27%; p = 0.17).

#### Anakinra

One poor-quality randomized discontinuation trial compared anakinra to conventional therapy among children with polyarticular, pauciarticular, or systemic JIA [8]. The main goal of the study was to evaluate safety. By week 28 of blinded treatment, 16% who received anakinra and 40% who received placebo had had a flare (p = 0.11). There was improvement in the CHAQ score in the anakinra group compared to placebo (-0.25 vs. 0.13; no p-value reported). Similarly, there was improvement in the ESR among those who were treated with anakinra (-2.21 vs. 13.73; no p-value reported). The quality was rated poor because the study did not have a sufficient sample size to assess efficacy and there was insufficient reporting of randomization and concealment.

#### Etanercept

Two studies evaluated etanercept versus placebo. One good-quality randomized discontinuation trial evaluated children with a polyarticular, pauciarticular, or systemic JRA [9]. In the double-blind component, fewer patients who received etanercept had a flare (28% vs. 81%; p = 0.003). There was also an improvement in the CHAQ score (-0.8 vs. -0.1). Overall, there was a 54% median improvement among those who received etanercept compared to no median change in the placebo group. There was an overall improvement in the number of active joints (7 vs. 13; no p-value reported), physician global assessment (2 vs. 5; no p-value reported), parent global assessment (3 vs. 5; no p-value reported), ESR (18 vs. 30; no p-value reported), and the ACR Pediatric 50 response (72% vs. 23%; no p-value reported).

The other study of etanercept was a fair-quality randomized controlled trial (RCT) that evaluated efficacy for the treatment of uveitis among children with JRA [10]. During the study, 6 of 12 subjects in the test treatment arm and 2 of 5 subjects in the conventional treatment arm improved. This was described by study investigators as no apparent difference.

#### Infliximab

One fair-quality RCT compared infliximab to conventional treatment among 121 children with polyarticular, pauciarticular, or systemic JRA [11]. The study did not find statistically significant differences between infliximab and conventional treatment in the ACR Pediatric 50 response at 14 weeks (50% vs. 33.9%, respectively; p = 0.13) or the rate of clinical remission at 52 weeks (44.1% vs. 43.1%, respectively).

#### Intravenous Immunoglobulin (IVIG)

Three studies compared IVIG to conventional treatment. One fair-quality randomized discontinuation trial [12] of 19 patients who had polyarticular JRA found a 3% decrease in the active joint count among those who were treated compared to a 30% increase in the placebo group. Physician global assessment improved for 3% of patients in the treatment group and worsened for 91% in the placebo group. Another, poor-quality study [13] compared IVIG to methylprednisolone among 20 subjects with JCA. Investigators found no statistically significant difference between the IVIG and methylprednisolone groups for ESR (59 at baseline and 21 at 6 months vs. 61 at baseline and 24 at 6 months, respectively). This study was rated poor because it was an open-label trial with no randomization, the subjects were incompletely described, and the analyses were not adjusted for baseline differences.

A poor-quality RCT [14] that included 31 subjects with systemic JRA found that IVIG compared to conventional therapy was associated with a non-statistically significant improvement in the median change in active joint count (-2 vs. -1) and in physician global assessment of improvement (50% improvement vs. 27% improvement; p > 0.3). This study was rated poor because the sample size was small, there was a high dropout rate.

#### Tocilizumab

One fair-quality randomized discontinuation trial of 43 subjects with JIA evaluated tocilizumab [15]. From the RCT component, the active joint count in the tocilizumab group decreased from 3.5 to 0. Similarly, in the conventional treatment group it decreased from 4 to 0. There was improvement in the CHAQ score for each group (-0.5 vs. -0.25). Both physician global assessment (51.0 to 5.5 vs. 51 to 14) and parent global assessment (51.0 to 4.5 vs. 55 to 39) improved. The ESR decreased for both the tocilizumab and conventional treatment group (35 to 0.1 vs. 38 to 15, respectively). The ACR Pediatric 70 response increased in the tocilizumab group from approximately 70% to approximately 80%, but decreased in the conventional treatment group from approximately 80% to approximately 30%.

### Non-biologic DMARDs vs. Conventional treatment with or without Methotrexate

#### Azathioprine

One fair-quality RCT evaluated azathioprine among 32 subjects with polyarticular-onset, pauciarticular-onset, or systemic-onset JRA [16]. At 16 weeks of treatment, this study found non-statistically significant improvements with azathioprine in the number of active joints (-7 vs. -1; *p *= 0.45), physician global assessment (-5 vs. -2; p = 0.12), and the proportion with 50% improvement in ESR (4/13 subjects vs. 2/11 subjects; p = 0.36).

#### Hydroxychloroquine

Two RCTs evaluated hydroxychloroquine. One good-quality trial of 162 subjects with polyarticular, pauciarticular, or systemic JRA (described in two publications [17,18]) found no significant difference in the change in mean active joint count compared to placebo after 12 months (6.7 [95% confidence interval (CI) -9.4 to -4] vs. -5.4 [95% CI -8 to -2.8]). The physician global assessment appeared slightly better for hydroxychloroquine than for placebo (70% better, 26% same, 2% worse compared to 53% better, 41% same, 6% worse; no p-value reported). There was no difference in the mean ESR decrease at 12 months (10 each).

The other study was a poor-quality, open-label RCT of 72 subjects with polyarticular or pauciarticular JRA that compared hydroxychloroquine to gold [19]. At 50 weeks, there were no statistically significant differences in the active joint count (-4 vs. -5), median change in the physician global assessment (-8 vs. -9), or change in the ESR (-12 vs. -11). Similarly, the physician overall assessment of at least 50% improvement was not statistically significantly different between the hydroxychloroquine group and the gold group (12 of 17 improved vs. 10 of 15 improved, respectively). This study was rated poor because allocation concealment was not specified, there were important differences in baseline characteristics, it was unclear if outcomes were assessed blinded to the intervention, and the outcomes were incompletely described.

#### Methotrexate

Three studies compared methotrexate to conventional treatment without methotrexate. One good-quality RCT of 127 subjects with JIA compared low-dose methotrexate, very low-dose methotrexate, and placebo in a 6-month trial [20]. The mean active joint count decreased with low-dose methotrexate (-7.5), very low-dose methotrexate (-5.2), and placebo (-5.2; p > 0.3 overall). Physician global assessment improved with low-dose methotrexate compared to placebo (p = 0.02), but there was no statistically significant difference between the low-dose and very low-dose methotrexate groups for this outcome (p = 0.06). Based on a composite index with at least 25% improvement in articular score and improvement according to physicians and parents, 63% of those in the low-dose methotrexate group improved, compare to 32% in the very low-dose methotrexate group, and 36% in the placebo group (p = 0.013).

Another good-quality study [21] of 88 subjects with extended oligoarticular or systemic JIA compared methotrexate to placebo among children with extended oligoarticular JIA or systemic JIA in a double-blind RCT with crossover. Among those with oligoarticular JIA, there was statistically significant improvement in physician global assessment (p < 0.001) and ESR (p < 0.001) with methotrexate. The change in the number of joints with synovitis (-3) did not achieve statistical significance (p < 0.1). Similarly, among those with systemic JIA, there was improvement in physician global assessment (p < 0.001), but not in ESR (p = 0.06) or in the number of joints with synovitis (p = 0.06) in patients taking methotrexate.

A poor-quality, non-randomized study that included 63 children with JIA compared methotrexate to NSAIDs and to methylprednisolone [22]. In this study, the active joint count improved more in the methylprednisolone group than in either the methotrexate or NSAID groups (-7.1 vs. -4 vs. -0.8, respectively; p = 0.008). This study was rated poor because there was confounding by indication, the analysis did not adjust for potential confounders, outcomes were not assessed blinded to treatment, and subjects were not blinded to treatment assignment.

#### Penicillamine

Four publications describing three distinct studies evaluated penicillamine. One good-quality RCT [17,18] among subjects with polyarticular, pauciarticular, or systemic JRA found no statistically significant effect on the mean active joint count with penicillamine compared to placebo after 12 months (-3 [95% CI -4.8 to -1.1] vs. -5.4 [-8 to -2.8]); results were similar for physician global assessment (56% better, 28% same, 16% worse vs. 53% better, 41% same, 6% worse) and mean decrease in ESR (9.4 vs. 10).

A fair-quality RCT [23] of 74 subjects with polyarticular-onset, pauciarticular-onset, or systemic-onset JCA found no statistically significant effect on ESR in a 6-month study in patients treated with penicillamine compared to conventional treatment (-18 vs. -8). However, this study did find a statistically significant decrease in the number of painful joints in patients taking penicillamine (-3 vs. -1.6; *p *< 0.04).

A previously described poor-quality, open-label RCT [19] that included 74 subjects with polyarticular or pauciarticular JRA found no statistically significant effect for penicillamine compared to gold at 50 weeks in the active joint count (-2.5 vs. -5), median change in the physician global assessment (-7.5 vs. -9), change in ESR (-8 vs. -11), or the proportion of patients who had at least a 50% improvement based on physician assessment (8/12 vs. 10/15).

#### Sulfasalazine

One good-quality RCT of 69 subjects with polyarticular or oligoarticular JCA evaluated sulfasalazine versus placebo [24]. In this study, it was unclear which time points were compared. However, there was statistically significant improvement with sulfasalazine in active joint count (-5.54 vs. -0.78; p = 0.005), physician global assessment (-1.95 vs. -0.99; p = 0.0002), patient/parent global assessment (-0.98 vs. -0.44; p = 0.01), and decrease in ESR (-0.74 vs. -0.04; p < 0.001). The number of improved joints by x-ray findings was not statistically significantly different (0.71 vs. 0.53).

## Comparative effects of different DMARDs (key question 2)

### Comparisons of biologic DMARDs

#### Etanercept vs. Infliximab

One poor-quality, non-randomized, open-label study compared etanercept to infliximab among subjects with polyarticular JIA [25]. Among the 10 patients receiving etanercept, one was withdrawn for non-compliance. Among the 14 patients receiving infliximab, 4 withdrew because of adverse events and 1 withdrew because of failure to reach the ACR Pediatric 50 response. After 12 months of treatment, the change in active joint count was similar between etanercept (-9.5 [95% CI -19 to -3]) and infliximab (-11.5 [95% CI -17 to -7.5]). Results were also similar in the two treatment groups for changes in the CHAQ score (-0.81 vs. -0.31; p = 0.12), physician global assessment (-29 vs. -35; p = 0.65), patient/parent global assessment (-24.5 vs. -27.5; p = 0.81), ACR Pediatric 75 (67% each), ACR Pediatric 50 (78% vs. 89%; p-value not reported, but calculated as 0.53), and ESR (28.5 vs. -25; p = 0.37). This study was rated poor because assessment was not described as blinded to treatment.

### Comparisons of non-biologic DMARDs

#### Penicillamine vs. Hydroxychloroquine

Two publications [17,18] described a good-quality RCT that compared penicillamine and hydroxychloroquine to placebo (results described above, under Key Question 1) and to one another. At 12 months, neither active drug was superior to the other based on active joint count, ESR, or physician global assessment.

One poor-quality, open-label RCT [19] compared hydroxychloroquine and penicillamine to gold (results described above, under Key Question 1) and to one another. At 50 weeks, there were no significant differences between the two DMARDs in active joint count, physician global assessment, or ESR.

#### Sulfasalazine vs. Hydroxychloroquine

One poor-quality RCT compared sulfasalazine to hydroxychloroquine in 39 subjects with oligoarticular-onset, polyarticular-onset, or systemic-onset JCA [26]. After 6 months, the average number of affected joints decreased by 1.5 in the sulfasalazine group and by 0.6 in the hydroxychloroquine group (no p-value reported). During this time, the ESR decreased in both the sulfasalazine group (52.7 to 36.3; no p-value reported) and hydroxychloroquine group (41.2 to 28.9; no p-value reported). Physician global assessment and patient global assessment were similar in the two groups. This study was rated as poor because there was incomplete description of the subjects and it was unclear if treatment and assessment were blinded.

#### Leflunomide vs. Methotrexate

One good-quality RCT compared leflunomide to conventional treatment with methotrexate in 94 subjects with polyarticular JRA [27]. This 16-week study with a 32-week blinded extension found improvements in both groups with no significant differences by treatment. The active joint count decreased for the leflunomide and conventional treatment groups (-8.1 vs. -8.9; p = not significant). Similarly, in both groups there were improvements in the CHAQ score (-0.44 vs. -0.39; p = not significant), physician global assessment (-31.5 vs. -32.1; p = not significant), parent global assessment (-15.9 vs. -22; p = not significant), and ESR (-6.5 vs. 7.2; p = not significant). As the trial proceeded, the methotrexate group appeared to have a greater improvement in the proportion of patients who had an ACR Pediatric 30, Pediatric 50, or Pediatric 70 response. For example, 70% of the leflunomide group and 83% of the methotrexate group achieved an ACR Pediatric 70 response at 48 vs. 16 weeks. The improvement was not statistically significant for either the leflunomide (p = 0.88) or methotrexate (p = 0.06) groups.

## Serious adverse events (key question 3)

The search identified 151 publications, including 19 RCTs, which reported adverse events possibly associated with a DMARD among patients with JIA. Although these reports described 4344 patients, there was insufficient information to determine whether some patients were included in more than one report. Furthermore, some series included patients who were adults or who did not have JIA. Thirteen of the 19 RCTs, representing 914 unique patients treated with 14 DMARDs or DMARD combinations, included placebo comparisons. With the exception of one patient who died 10 days after receiving a placebo infusion but who had also received methotrexate, none of these RCTs reported incident cases of either cancer or death among patients receiving a DMARD during the RCT phase of the study.

Of the 4344 (potentially not unique) patients represented in the eligible studies that reported adverse events, 11 incident cases of cancer were reported. One case of thyroid carcinoma was associated with etanercept [28], one case of thyroid carcinoma was associated with etanercept plus methotrexate [29], and one case of yolk sac carcinoma was associated with etanercept plus methotrexate [29]. The remaining eight incident cases of cancer were lymphomas: two cases with etanercept plus methotrexate [29,30]; two cases in patients who had received infliximab, etanercept, and methotrexate [30]; three cases with methotrexate alone; and one case with methotrexate and cyclosporine A, which was diagnosed at autopsy after death attributed to Legionella pneumonia [31-34]. Insufficient data are available to assess the impact of duration of treatment on the risk of having a serious adverse event.

## Efficacy, effectiveness, safety, and adverse effects across different diagnostic categories of JIA (key question 4)

One study compared the efficacy of the DMARD studied (methotrexate) across different diagnostic categories of JIA [21]. There was no statistically significant difference in the efficacy of methotrexate for oligoarticular JIA versus systemic JIA. No data on adverse events was provided.

## Discussion

Few data are available to evaluate the comparative effectiveness of DMARDs. Methotrexate is the most studied DMARD and good-quality studies support its efficacy. The paucity of evidence precludes direct comparisons of the other, newer DMARDs against each other.

Research on the effectiveness of treatments for JIA is challenging because it includes multiple categories that could potentially respond differently to therapy. Furthermore, the health impact of JIA fluctuates over time. Despite this, our review found that based on the overall number, quality, and consistency of studies, there is moderate strength of evidence to support that DMARDs improve symptoms associated with JIA. However, the strength of evidence is low that DMARDs improve overall health status.

There was significant variation in how outcomes were reported. For example, among the six randomized discontinuation trials, four reported laboratory measures of inflammation [6,8,9,15], four reported whether a flare occurred [6-9], three reported active joint count [6,12,15], and four reported quality of life based on the CHAQ [6,8,9,15]. Of those that reported CHAQ score, one [9] reported only the percentage change from baseline, and two [8,15] gave only average values without measures of dispersion. Standardizing outcome measures used in studies would allow for direct comparisons and patient-level meta-analysis. Ideally, these outcome measures should be clinically relevant and feasible to measure in both research and non-research settings. Such measures would help patients, families, and healthcare providers evaluate treatment options.

This review identified the important need for trials evaluating the effectiveness of DMARDs versus both conventional therapy and other DMARDs across categories of JIA. Factorial designs involving multiple treatments are a potential solution to challenge of low sample sizes in studies of rare conditions. In addition, patient-level meta-analysis of treatment outcomes could increase sample sizes, but only if trials are designed to include similar outcome measures.

There is a lack of information on adverse events associated with DMARDs in children with JIA. However, our findings suggest that short-term mortality associated with DMARDs is low. Because adverse events may not occur in the short time periods used in drug trials, the development of registries of patients treated with DMARDs may be necessary to accurately assess both risks and benefits [35]. Until such registries are available, assessment of other sources of data will be important [35]. For example, a recent report that combined a review of the literature with drug company-sponsored post-marketing database suggests that the risk of any malignancy with etanercept, regardless of indication, is about 0.02 per 100 patient-years among patients 4 to 17 years of age, compared to 0.015 among the general population of children 4 to 17 years of age in the United States [36]. Of course, patients, families, and physicians will need to make important treatment decisions before these new data systems are available.

## Conclusions

JIA is an important cause of morbidity. Few data are available to evaluate the comparative effectiveness of either specific DMARDs or general classes of DMARDs. Moderately strong evidence supports that DMARDs improve the symptoms associated with JIA. Limited data suggest that short-term risk of cancer is low. To support shared decision-making around the use of DMARDs based on these findings, educational material for patients and families [37] and clinicians [38] has been developed by AHRQ.

## Abbreviations

ACR: American College of Rheumatology; AHRQ: Agency for Healthcare Research and Quality; CER: Comparative effectiveness review; CI: Confidence interval; CHAQ: Childhood Health Assessment Questionnaire; DMARD(s): Disease-modifying antirheumatic drug(s); ESR: Erythrocyte sedimentation rate; FDA: U.S. Food and Drug Administration; IVIG: Intravenous immunoglobulin; JCA: Juvenile chronic arthritis; JIA: Juvenile idiopathic arthritis; JRA: Juvenile rheumatoid arthritis; MeSH: Medical Subject Headings; NSAIDs: Non-steroidal anti-inflammatory drugs; RCT: Randomized controlled trial; TNF: Tumor necrosis factor.

## Competing interests

The authors declare that they have no competing interests.

## Authors' contributions

ARK, JWW, and GDS conceived and designed the review. ARK, RRC, JWW, and GDS developed the search strategy and the data abstraction forms. All authors reviewed the articles, abstracted data, and participated in the data synthesis. ARK drafted the manuscript, with critical review by HAQVM, RRC, JWW, and GDS. All authors read and approved the final manuscript.

## Pre-publication history

The pre-publication history for this paper can be accessed here:

http://www.biomedcentral.com/1471-2431/12/29/prepub
